# Loot

**Published:** 1868-12-15

**Authors:** 


### LOOT.

### *American and Foreign.*

Prof. Gross, in the course of his eloquent response, on his second reception, referred to in the last number of the JOURNAL, observed:

"I need not say how deeply sensible I am of your kindness. I rejoice to be again in the midst of those with whom I have so long labored to uphold the honor and dignity of our noble profession, and in whose personal success I shall ever feel a deep, nay, let me add, a parental interest. It is to me no less gratifying than it is true, to be able to say that, during my visit abroad, where I had an opportunity of seeing many of our most distinguished brethren in the Old World, I saw no more able, learned, or skillful practitioners, teachers, and writers, than are assembled here to-night. I think, sir, that if a traveler learned nothing more than to appreciate fully his country's greatness, he would be amply compensated for the peril and expense of his voyage across the Atlantic. As God made woman more beautiful and perfect than man, because he created her last, so he endowed this continent — this last and best gift to the human race — with beauty and perfection nowhere visible in the Old World.

"In our profession an American traveler finds nothing of any importance, that is either new or unknown here. Every one acknowledges, with a hearty, free will, the extraordinary activity, enterprise, and talents of our physicians and surgeons, and the rapid strides with which we are developing our national medical talent. Chassaignac, the great French surgeon, said to my honored colleague and myself, in one of our visits to the famous Lariboisier Hospital, "You have just reason to be proud of your country. America at this moment wields the surgical sceptre of the world." Her military surgeons have no equals. The reports of the Surgeon-General of the United States are every where read with avidity. Some of our works are used as text-books in the British schools of medicine, and a number of them have been translated into the continental languages. It is amusing how small and scanty are the libraries of many of the medical celebrities of Vienna, Berlin, London, Edinburgh, Dublin, and other cities, in comparison with those of similar professional standing here; and, although it would not be fair to judge a man's knowledge by the number of books he reads, yet there can be no doubt that the more labor of this kind he performs, the more likely will he be to become intelligent and accomplished. In no country that I saw, do people read so much as here. It is a well-known fact, that many of the English medical works, reprinted in America, pass through more editions than they do at home."

W. M. Cornell, M.D., LL.D., in the *Guardian of Health*, discourses thus on the *Treatment* of Apoplexy:

The common treatment in an attack of apoplexy, is to bleed profusely from the arm or the head, then place the patient on a seat, with his feet in a tub of hot water, and to apply ice to the head, or pour upon it a stream of *cold* water from some height. With this treatment they nearly all die — that's all. Yet it has been that usually pursued for three thousand years, and it is difficult to get out of it. A *better* treatment was pointed out, some twenty years ago, by Dr. Samuel A. Cartwright, of New Orleans, and published in the "Medical and Surgical Journal" of that city, which Dr. C. says, "fell still-born from the press." I may add, like all other notions which conflict with "the books," and the general *routine* practice. Still, the writer has followed it during more than twenty years, and has not lost a patient with ordinary apoplexy, or that from *sun-stroke.*

Dr. Cartwright said, "my theory of apoplexy is nearly the same as that of Marshall Hall's; but my practice is much better and easier. For twenty-five years I have been in the habit of curing apoplexy almost as readily as intermittent fever. Physicians will not avail themselves of the practice, because the old theory of apoplexy will not let them. The laryngismus and trachælisimus, that Hall speaks of, can be cured by a mixture of salt, mustard, ipecac, and tincture of assafœtida, put into the mouth and fauces very speedily. Patients can swallow that, when the deglutition of plain water is impossible. If the stertor or ronchus be very great, with stupor corresponding to the stertor and relaxation of the spinchters, I add capsicum, quinine, and laudanum, in full doses, to the mixture. At first it strangles, but by turning the patient on his side, and discharging the throat of the tough mucus or phlegm, *always in it* in such cases, and trying again and again, the patient will soon be able to swallow enough to vomit. If this be assisted by hot water to the head and stomach, the patient will soon regain his senses, after having taken enough to vomit. In my essay on apoplexy, I was afraid of spoiling it, by saying much on hot water, as the medicine above mentioned will do in mild cases; but *coup de soleil* can not be cured without the hot water."

Now, this was published in the "Boston Medical and Surgical Journal," more than twenty years ago, and, as said above, the writer has tried it successfully all this time. But he does not know of a single other physician who has. They have all tried the bleeding and the *ice*-treatment, and they have nearly all died. Thus once has the writer stepped aside from a mere *Guardian* of Health, to tell how to *restore* it in apoplexy or sun-stroke.

To avoid apoplexy, live so as not to grow too fat, shun all employment that compels you to stoop much, take chiefly a vegetable diet, lead a quiet life, and, as Dr. Abernethy used to tell his dyspeptic patients, "live like a Christian." If you have been in active business, continue it. This is of the utmost importance. Read Milton's Epitaph on the death of old Hobson, the University carrier, or old Parr's death at the Palace. Like them, many die when just beginning to *live easy; i. e.*, living easy kills them quickly:

"Here lies old Dobson; Death has broke his girt,
And here, alas! hath laid him in the dirt;
Time's such a shifter, that if truth were known,
Death was half glad when he had got him down,
And surely death could never have prevailed,
Had not his weekly course of carriage failed.
Here lieth one, who did most truly prove
That he could never die while he could move.
Time numbers motion; yet, without a crime
'Gainst old truth, motion numbered out his time.
Rest that gives all men life, gave him his death,
And too much breathing put him out of breath."

This is the case with multitudes. Having nothing to do but eat and fatten, they soon die.

### *Statistics of Inebriation.*

Dr. McKinley, of this city (St. Louis), furnishes the following table of Statistics on Inebriation in the United States, which have been compiled by him after much careful research, and contain some very interesting yet startling facts. He says:

Taking the population of this country at forty millions, we find that of 300 men, 122 never drink ardent spirits at all, 100 drink moderately, but not to intoxication, 50 are ephemeral drinkers, 25 drink periodically, called "spreeing," and 3 are habitual inebriates. To every 178 who drink, 3 are confirmed inebriates, 25 are periodical drinkers, 50 are ephemeral drinkers, and 100 are moderate drinkers. Total, 178; non-drinkers, 122. Grand total, 300. One confirmed inebriate to every 59⅓ of men. Of 700 women, 600 never taste alcoholics of any kind, 30 taste wine occasionally, 17 taste ardent spirits occasionally, 36 drink beer or ale constantly, 14 drink ardent spirits periodically, and 3 are habitual inebriates. To every 100 who drink, 3 are confirmed inebriates, 14 drink ardent spirits periodically, 36 drink beer or ale constantly, 17 taste ardent spirits, and 30 taste wine occasionally. Total, 100; non-drinkers, 600. Grand total, 700. Total of both sexes enumerated — drinkers and non-drinkers — 1,000; total of both sexes who drink (out of 1,000), 278. Predominance in *confirmed inebriates* of the sexes: 3 men in every 178; 3 women in every 100; 1 confirmed inebriate to every $33\frac{1}{3}$ of women.

Fewer women drink than men; but a larger proportion of them become habitual drinkers.

The following deductions are made from the above statistical basis, and relate to the aggregate population of adult males and females, *using* and *not using* alcoholic drinks: In 1,000 men there are 10 confirmed inebriates; in 1,000 men, there are $593\frac{1}{3}$ drinkers of all kinds, who drink to some extent or other.

Of women — 1 in every 7 use alcoholics in some form; in 1,000 women, there are $142\frac{6}{7}$ who drink to some extent; in 1,000 women, $4\frac{2}{7}$ are confirmed inebriates.

Debauch or ephemeral drinkers, rarely become habitual, but periodical drinkers; the latter rarely become habitual inebriates, as the violence of their drinking is too great, and leads to disgusting satiety, and hence to intervals of sobriety. The moderate drinkers form the class from which the habitual inebriates are chiefly derived; prolonged moderate drinking cultivates the diathesis. The foregoing statistics are drawn from the seggregational population, including small and large towns, but excluding the gregarious population of cities, where the ratio of drinkers is higher. — *St. Louis Medical Reporter.*

### *Vaginal Irritability.*

At the April meeting of the Paris Medical Society, M. Charrier described the following case:

In September, 1867, a young woman was sent to him, who, after four months of married life, could no longer endure coitus. Copulation, at first excessive, became by degrees so painful, that she compares it with a severe toothache; indeed, she fainted occasionally, when the pain was for a moment suspended.

*Ext. Belladonna*, *Ext. Opii*, tepid baths, etc., were tried without benefit.

On examination, Charrier found the hymen ruptured, the myrtiform carunculæ not unusually large, no vulvitis, no vaginitis, no dryness of vulva or vagina; shrieks of pain were elicited by touching the carunculæ with the end of a sound. The least touch of the same with the tip of a finger, or even with a feather, produced the same effect. An attempt to pass the little finger into the vaginal orifice was resisted by strong contractions, similar to those met with in examinations for fissured anus.

Charrier instituted the following treatment: After a bath of two hours duration, she was placed thoroughly in anæsthesia; then the bladed speculum (for the removal of polypi), was introduced into the vagina, by a screw-like movement, and turned in every direction, as in the forcible dilatation of the anus. Some blood appeared. Then a very thick tent, (mèche), covered with extract of belladonna and glycerin, was introduced.

That night coitus took place without pain, and for two weeks it was repeated morning and evening. The husband had since written that his wife remained free from pain, and that she is now three months with child.— *Algem. Med. Central Zeitung*, No. 58, 1868.—*Medical and Surgical Reporter.*

